# Acute respiratory distress syndrome associated with macrophage activation syndrome in systemic lupus erythematosus: A case report and literature review

**DOI:** 10.1097/MD.0000000000028612

**Published:** 2022-02-04

**Authors:** En-Shuo Chang, Han-Hua Yu, Chiao-En Wu, Tien-Ming Chan

**Affiliations:** a Division of Nephrology, China Medical University Hsinchu Hospital, Hsinchu, Taiwan; b Division of Rheumatology, Allergy and Immunology, Chang Gung Memorial Hospital, Linkou branch, Taoyuan, Taiwan; c Division of Hematology-Oncology, Department of Internal Medicine, Chang Gung Memorial Hospital, Linkou branch, Taiwan; d Chang Gung University College of Medicine, Taoyuan, Taiwan.

**Keywords:** acute respiratory distress syndrome, case report, early diagnosis, macrophage activation syndrome, systemic lupus erythematosus

## Abstract

**Rationale:**

Previous treatment for macrophage activation syndrome (MAS) includes high-dose intravenous methylprednisolone along with intravenous immunoglobulin G. If MAS worsened, second-line therapy consisted of anakinra; if the disease remained refractory, third-line therapy with etoposide was considered. In addition, cyclosporine A plays a role in early MAS and in preventing recurrence. Some studies have reported the use of cytokine-targeting agents other than anakinra, such as canakinumab, tocilizumab, abatacept, and tofacitinib.

**Patient concerns:**

The patient with systemic lupus erythematosus (SLE) had an uncommon combination of intermittent fever, hyperferritinemia, hypertriglyceridemia, jaundice, and significantly abnormal liver function test results. The patient reported a history of daily fever of 38 to 39°C, painful oral ulcer, anorexia, abdominal bloating, diarrhea, and malar rash progression for 2 weeks, and jaundice, tea-colored urine, and clay-colored stool for 1 week preceding hospital admission.

**Diagnosis:**

SLE flareups in the patient were initially suspected. However, the final diagnosis was acute respiratory distress syndrome (ARDS) associated with MAS.

**Interventions:**

The treatment included disease-modifying antirheumatic drugs (DMARDs), such as azathioprine, and titrated steroid doses of methylprednisolone (40 mg q8 h) and dexamethasone (15 mg q8 h), after the patient had ARDS and was intubated.

Dose-adjusted monotherapy with dexamethasone was found to be effective; this may be attributed to some DMARDs being unsuitable for cytokine storms, that is, some DMARDs may cause complications in cytokine storms.

**Outcomes:**

After dexamethasone 15 mg q8 h treatment, the patient's fever subsided within 2 days, and liver function became normal within 3 weeks. The patient regularly attended scheduled outpatient follow-up visits after discharge. After 2 years, the patient reported no symptoms or signs of SLE with 2 mg/d oral dexamethasone.

**Lessons:**

Early diagnosis of MAS and dexamethasone treatment for MAS with ARDS appear to be crucial for these patients.

## 1 Introduction

Macrophage activation syndrome (MAS) is a life-threatening form of hemophagocytic lymphohistiocytosis (HLH) secondary to autoimmune disease caused by uninterrupted immune cell hyperstimulation.^[1,2]^ Diagnosis can be difficult because of its nonspecific clinical characteristics, including persistent high-grade fever, hepatosplenomegaly, lymphadenopathy, hemorrhagic manifestations, and sepsis-like features. Delayed diagnosis and treatment leads to high morbidity and mortality rates. Diagnostic criteria have been proposed for HLH and MAS associated with systemic juvenile idiopathic arthritis and systemic lupus erythematosus (SLE).^[2]^ previously reported treatments for MAS include etoposide, dexamethasone, and cyclosporine. We report a 26 year-old woman with SLE who developed MAS and acute respiratory distress syndrome (ARDS), and was successfully treated with dexamethasone monotherapy. Early diagnosis and dexamethasone treatment appear to be critical in these patients.

## 2 Case report

A 26 year-old East Asian woman with a history of reflux esophagitis (Los Angeles grade A) and SLE complicated by lupus encephalopathy initially presented to the Chang Gung Memorial Hospital with a seizure episode. The patient reported a daily fever of 38 to 39°C, painful oral ulcers, anorexia, abdominal fullness, diarrhea, and malar rash progression over the preceding 2 weeks and a 1 week history of jaundice, tea-colored urine, and clay-colored stool. At a recent outpatient visit, her symptoms were attributed to an SLE flare (leukopenia, decreased C3 level, elevated anti-dsDNA level, and fever without infection), and her dose of methylprednisolone was titrated to 30 mg/d. Physical examination revealed icteric sclera, bilateral basilar crackles, and epigastric tenderness. Because of persistent jaundice, she was admitted to the hospital with an acute SLE flare. Azathioprine and ezetimibe/simvastatin were discontinued, hydroxychloroquine was continued (200 mg/d [previous long-term use]), and methylprednisolone was increased to 80 mg/d.

Laboratory testing at admission showed pancytopenia, hypoalbuminemia, conjugated hyperbilirubinemia (total, 8.7 mg/dL; conjugated, 5.0 mg/dL) and elevated levels of alanine aminotransferase (ALT) (277 U/L), aspartate aminotransferase (AST) (809 U/L), alkaline phosphatase, and lipase (292 U/L). Immunological testing showed elevated anti-dsDNA level (295.2 IU/mL) and low C3 level (42.9 mg/dL). The C4 level was within the normal range (12.8 mg/dL). Tests for viral hepatitis, mitochondrial and smooth muscle antibodies, immunoglobulin G, and hepatitis B surface antigen were unremarkable. Viral testing for influenza A/B, herpes virus, Epstein–Barr virus, cytomegalovirus, and human immunodeficiency virus, and a rapid plasma regain test were negative. Legionella antigen was not detected. Sputum, urine, and blood cultures revealed no aerobic or anaerobic growth. Hemoglobin level was 9.3 g/dL, platelet count of 44,000 μL, and white blood cell count of 2500. His prothrombin time was 11.5 seconds (INR 1.1).

Ischemic hepatitis was ruled out due to the absence of septic or cardiogenic shock, and drug-related hepatitis (including azathioprine) was excluded from the study. Abdominal computed tomography revealed mild swelling of the pancreas with retroperitoneal fat stranding, suggestive of pancreatitis. No evidence of hepatomegaly, splenomegaly, or biliary obstruction was found.

After the seizure on the second day of admission, intravenous methylprednisolone (80 mg/d) was initiated. One week later, laboratory testing showed increased levels of bilirubin (total, 13.8 mg/dL; conjugated, 7.9 mg/dL), AST (1260 U/L), and ALT (567 U/L) and prolonged prothrombin time (22.9 seconds, INR 2.1). Immunological testing revealed decreased levels of anti-dsDNA (154.9 IU/mL), C3 (23.5 mg/dL), and C4 (6.53 mg/dL). The patient was subsequently transferred to the intensive care unit because of acute progression of hepatitis.

**Figure 1:**
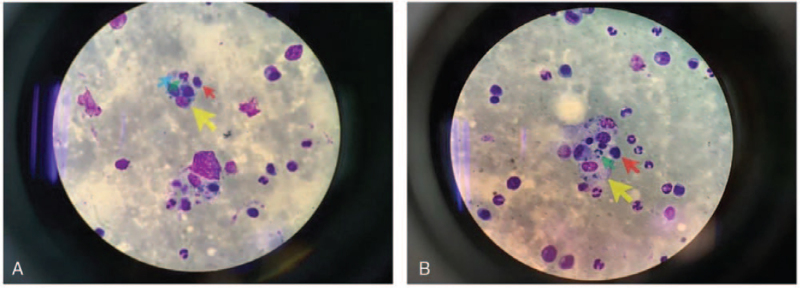
Bone marrow smear shows (A) hemophagocytosis of a lymphocyte (red arrow), a neutrophil (green arrow), and several platelets (blue arrow) by a histiocyte (yellow arrow) and (B) hemophagocytosis of a neutrophil (green arrow) and several lymphocytes (red arrow) by a histiocyte (yellow arrow).

After transfer to the intensive care unit, the patient experienced acute-onset respiratory distress, and intubation was performed because of hypoxic respiratory failure. ARDS was diagnosed on the basis of the Berlin definition (low P/F ratio and diffuse bilateral lung infiltrates). Laboratory testing in the intensive care unit showed hyperferritinemia (6952 mcg/L), hypertriglyceridemia (395 mg/dL), anemia (7.7 g/dL), severe thrombocytopenia (6000/μL), and hypofibrinogenemia (121 mg/dL). To rule out hematologic disease, we ordered a peripheral blood smear, which showed thrombocytopenia with red blood cell fragmentation and consulted a hematologist. Since MAS was highly suspected due to high fever without an infection focus, bone marrow aspiration and biopsy were performed. Bone marrow examination revealed proliferation of histiocytes with hemophagocytosis (Fig. 1). This confirmed the diagnosis of MAS presenting as HLH secondary to SLE, based on the 2004 HLH diagnostic criteria (Table 1).^[3]^ The patient also met the H-score criteria for diagnosis of reactive hematophagocytic syndrome (Table 2).

**Table 1:** Diagnostic criteria for macrophage activation syndrome.

Parameter	Our patient	Yes/No
Clinical criteria		
(1) Fever (>38°C)	Fever of 39.7°C (day 15)	Yes
(2) Hepatomegaly (≥3 cm below the costal arch)	No hepatomegaly on abdominal CT	No
(3) Splenomegaly (≥3 cm below the costal arch)	No splenomegaly on abdominal CT	No
(4) Hemorrhagic manifestations (purpura, easy bruising, or mucosal bleeding)	No associated symptom/signs	No
(5) Central nervous system dysfunction (irritability, disorientation, lethargy, headache, seizures, or coma)	Seizure (day 2)	Yes
Laboratory criteria		
(1) Cytopenia affecting 2 or more cell lineages (white blood cell count ≤4.0 × 10^9^/L, hemoglobin ≤90 g/L, or platelet count ≤150 × 10^9^/L)	White blood cell count 2000/μL (day 5), hemoglobin 83 g/L (day 7), platelet 6000/μL (day 9)	Yes
(2) Increased aspartate aminotransferase (>40 U/L)	AST 1357 U/L (day 8)	Yes
(3) Increased lactate dehydrogenase (≥567 U/L)	N/A	No
(4) Hypofibrinogenemia (fibrinogen ≤1.5 g/L)	Fibrinogen 112 mg/dL (day 9)	Yes
Hypertriglyceridemia (>178 mg/dL)	Triglyceride 395 mg/dL (day 11)	Yes
(5) Hyperferritinemia (ferritin >500 mg/L)	Ferritin 695.2 μg/L (day10)	Yes
Histopathologic criterion		Yes
Evidence of macrophage hemophagocytosis in bone marrow aspirate	Bone marrow exam showed hemophagocytosis	

AST = aspartate aminotransferase, CT = computed tomography, N/A = not applicable.

**Table 2:** H-score for reactive hematophagocytic syndrome in our patient.

Parameter	Our patient	H-score
Clinical criteria		
Fever	Fever of 39.7°C (day 15)	49
Hepatomegaly	No hepatomegaly on abdominal CT	0
Splenomegaly	No splenomegaly on abdominal CT	0
Immunosuppression	Prednisolone therapy	18
Laboratory criteria		
Cytopenia in 2 or 3 lineages	Hemoglobin 8.3 g/dL (day 8), platelet 6000/μL (day 10), WBC 2000/μL (day 6)	34
Ferritin, ng/mL	Ferritin 6952 ng/mL	50
Hypertriglyceridemia, mmol/L	Triglyceride 395 mg/dL (day 11)	64
Hypofibrinogenemia, g/L	Fibrinogen 112 mg/dL (day 9)	30
Liver function tests, IU/L	AST 1357 U/L (day 8)	19
Hemophagocytosis	Bone marrow exam revealed hemophagocytosis	35
Total score (score >169 is 93% sensitive and 86% specific)		299

AST = aspartate aminotransferase, CT = computed tomography, WBC = white blood cells.

**Figure 2:**
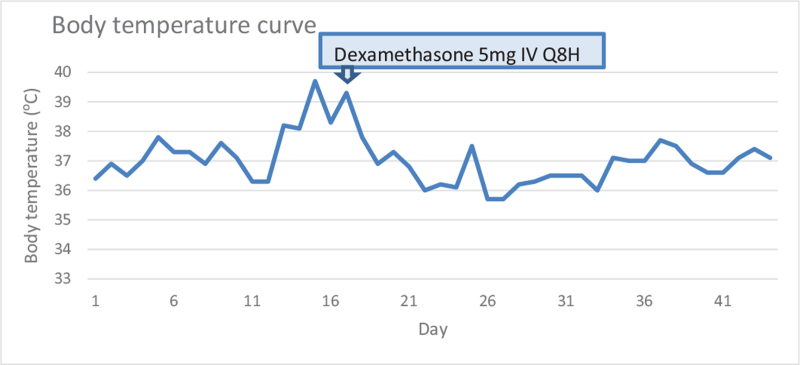
Body temperature during admission.

A rheumatologist suggested dexamethasone titration. We discontinued methylprednisolone and initiated intravenous dexamethasone (15 mg/d). Two days later, the fever subsided significantly (Fig. 2) and the patient was extubated. Liver function test parameters returned to nearly normal within 3 weeks. The patient regularly attended scheduled outpatient follow-up visits after discharge. After 3 years, she reported no symptoms or signs of lupus activity while taking oral dexamethasone (2 mg/d).

## 3 Discussion

MAS is a secondary HLH that can be a fatal complication of SLE. MAS-induced cytokine storms may lead to multiple organ failure. The most frequently involved organs are the liver and spleen; hepatomegaly, splenomegaly, and abnormal liver function tests may result.^[1]^ Pulmonary involvement has been reported as ARDS that requires mechanical ventilation support.^[2]^ Gastrointestinal bleeding, pancreatitis, or ulcerative colitis indicate gastrointestinal involvement. Neurological presentations include coma, meningitis, or seizures, whereas renal involvement is indicated by evidence of acute kidney injury or nephrotic syndrome.^[1]^

Distinguishing SLE flares from MAS can be challenging. MAS should be considered in patients with autoimmune disease and fever of unknown origin, liver dysfunction, or peripheral cytopenia.^[1,4]^ The H-score is superior to the 2004 HLH diagnostic criteria for diagnosing secondary HLH.^[5]^ The H-score in the patient presented here was 299, which exceeded the diagnostic cutoff of 169 (Table 2). Poudel et al^[6]^ reported a patient who presented with MAS as the initial manifestation of SLE, and concluded that early diagnosis is crucial because mortality is high in untreated cases.

The 2004 HLH protocol is the standard treatment for primary HLH; however, no guidelines exist for MAS treatment. Nonetheless, Carter et al^[2]^ have recommended an MAS treatment protocol: first-line therapy is high-dose intravenous methylprednisolone 1 g/d for 3 to 5 days plus intravenous immunoglobulin G 1 g/kg/d for 2 days. If MAS deteriorates, second-line therapy consists of anakinra, ranging from 1 to 2 mg/kg/d and up to 8 mg/kg/d. If the disease remains refractory, discussion of treatment with a hematologist and third-line therapy is recommended. Third-line therapy consisted of etoposide 150 mg/m^2^ twice weekly for 2 weeks, followed by once weekly for 6 weeks. In addition, cyclosporine A at 2 to 7 mg/kg/d acts as a parallel treatment for early MAS or for preventing recurrence.^[2]^

Some studies have reported cytokine-targeting agents other than anakinra, including canakinumab, tocilizumab, abatacept, and tofacitinib.^[7]^ Another study reported that the 2004 HLH protocol may be useful when a patient is unresponsive to steroids and cyclosporine A.^[8]^ In addition to medical therapy, plasmapheresis, in combination with immunosuppressive therapy, is an option.^[9]^ We successfully treated our patient using only dexamethasone plus previous long-term hydroxychloroquine. Although Nakagishi et al^[10]^ previously reported the successful use of dexamethasone palmitate to treat MAS, it was used in addition to methylprednisolone pulse therapy or tocilizumab with cyclosporine A.

Steroid monotherapy with intravenous dexamethasone up to 15 mg/d may be an effective and simple regimen for MAS complicated by ARDS. The reason for successful adjusted monotherapy with dexamethasone may be attributed to some disease-modifying antirheumatic drugs (DMARDs) being unsuitable for cytokine storms. Some DMARDs cause cytokine storms to become more complex. Hydroxychloroquine is the mildest immunomodulator, and numerous studies have shown that its benefit is greater than its toxicity. Therefore, we continued HCQ (200 mg 1 pc QD) as baseline control even after we thought that there were no other DMARDs suitable at that time. Dexamethasone is more durable than methylprednisolone and should be administered at high doses when an infection is not suspected. The case presented here illustrates that its use can result in the improvement of abnormal liver function, jaundice, and ARDS.

## 4 Conclusion

In conclusion, we suggest that early diagnosis of MAS is critical; a high degree of suspicion is needed, and laboratory testing for ferritin, triglycerides, and bone marrow aspiration may be required. Dexamethasone administration is important to avoid cytokine storms and can successfully treat ARDS attributed to MAS associated with SLE.

## Acknowledgments

We would like to thank all doctors and nursing colleagues of the Gastroenterology Intensive Care Unit, Department of Rheumatology and Immunology, and Department of Hematology and Oncology who take care of patients. We would also like to thank the patient and her family for their strong support in this work, for providing us with all relevant information, and for their willingness to publish the entire disease process of the patient to help doctors manage this difficult situation and ensure adequate medical services in the future.

## Author contributions

**Conceptualization:** Tien-Ming Chan.

**Data curation:** En-Shuo Chang, Han-Hua Yu.

**Supervision:** Tien-Ming Chan, Chiao-En Wu.

**Visualization:** Han-Hua Yu.

**Writing – original draft:** En-Shuo Chang.

**Writing – review & editing:** Tien-Ming Chan.
